# Evaluating 3D-printed models for congenital heart disease: impact on parental anxiety and procedural understanding

**DOI:** 10.1038/s41390-025-03999-x

**Published:** 2025-03-17

**Authors:** Linda Grefen, Felix Rudolf, Christopher Herz, Nikolaus A. Haas, André Jakob, Christian Hagl, Paul Philipp Heinisch, Jürgen Hörer, Nikolaus Thierfelder, Maximilian Grab

**Affiliations:** 1 Department of Cardiac Surgery, LMU University Hospital, LMU Munich, Munich, Germany; 2 German Centre for Cardiovascular Research (DZHK), Partner Site Munich Heart Alliance, Munich, Germany; 3 Chair of Medical Materials and Implants, Technical University, Munich, Germany; 4 Department of Pediatric Cardiology and Intensive Care, LMU University Hospital, LMU Munich, Munich, Germany; 5 Department of Congenital and Pediatric Heart Surgery, German Heart Center Munich, Technical University Munich, Munich, Germany; 6 Division of Congenital and Pediatric Heart Surgery, LMU University Hospital, Munich, Germany

## Abstract

**Background:**

Evaluation of the impact of 3D-printed models on parental education and anxiety in parents of children with congenital heart disease (CHD) who are undergoing interventions or surgeries.

**Methods:**

A prospective randomized controlled trial was conducted at LMU University Hospital and the German Heart Center Munich. Parents (*n* = 57) of pediatric CHD patients were randomized into two groups: a control group using standardized paper-based methods and an intervention group using additional 3D-printed models. Parental anxiety was assessed using the Visual Analog Scale (VAS) and the State-Trait Anxiety Inventory, while procedural understanding and satisfaction with the education were evaluated using custom-developed questionnaires.

**Results:**

Both educational methods significantly increased procedural understanding (control 77.5% to 92.3%; 3D model: 77% to 89.4%, *p* < 0.0001, respectively). Significant reduction of anxiety was observed in both groups, (VAS reduction control: −0.9, *p* = 0.0342; 3D model: −1.1, *p* = 0.0116). Parents with medical background or those informed by pediatricians had lower anxiety whereas those seeking information online exhibited higher anxiety levels.

**Conclusion:**

Both educational methods significantly reduced parental anxiety and improved procedural understanding. 3D-printed models were well-received and will be integrated into routine practice to enhance education on CHD procedures and optimize physician-parent communication.

**Impact Statement:**

- Using additional 3D-printed models of congenital heart defects significantly reduces parental anxiety and increases procedural understanding of complex interventions or surgeries.
- Physicians are in desperate need of new visualization methods for an efficient patient education.
- This study underscores the need for tailored educational and psychological support for parents based on their previous experiences.

## Introduction

Congenital heart defects (CHD) are the most common congenital anomaly, affecting about 1% of live births in Germany.^1^ These defects range from simple conditions that may resolve independently to complex malformations requiring multiple surgeries or interventions. Despite advances in CHD treatment and improved outcomes, the psychological burden on parents remains significant.^2^ Studies show that interventions and surgeries for CHD are major sources of parental anxiety, compounded by hospitalization and complex, high-risk procedures.^3,4^ Parents of hospitalized children often experience heightened feelings of helplessness, fear and anxiety.^5^ The anatomical and procedural complexity of CHD further complicates obtaining patients’ or parents’ informed consent for the intervention or surgery. Patient education (PE) is commonly performed using pre-printed paper-based methods. Even though physicians dealing with CHD already have a strong tendency to add detailed drawings of the pathologies, parents with no previous knowledge may still have trouble following the procedural steps. Studies performed with adult patients in various medical fields already showed a significant reduction of preoperative anxiety when using anatomical models, augmented and/or virtual reality or video sequences.^6–8^ The European Pediatric Heart Center deals with the most complex cases of CHD, where parents are often confronted with multiple complex procedures. The aim of this study was therefore to investigate the possible impact of using additional 3D-printed models for the parental education of patients with CHD, both interventional and surgical, with the evaluation of a subsequent reduction in anxiety at our center.

## Materials and methods

### Study design

**Fig. 1:**
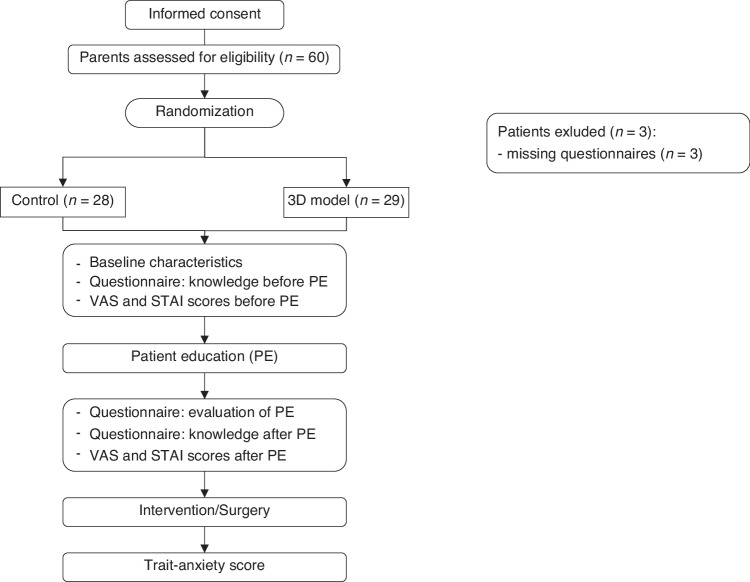
Overview of study design and randomization. After informed consent, parents were randomized and completed the described questionnaires at different timepoints.

A prospective randomized controlled trial (ISRCTN registry number: ISRCTN71913555) was conducted from January 2022 to May 2024 at LMU University Hospital and the German Heart Center Munich (European Pediatric Heart Center, EKHZ). A total of 60 parents of pediatric patients were randomized (3 questionnaires incomplete) to either a control group using standardized paper-based methods (*n* = 28) or a 3D-printed model group (*n* = 29) (Fig. 1). After excluding incomplete questionnaires, 57 participants remained. Informed consent was obtained, and all data were pseudoanonymized. The study received ethical approval (LMU project number 22-0281). Parents of pediatric patients with CHD (e.g., septal defects, tetralogy of Fallot, coarctation of the aorta) were included. Exclusion criteria included prior education by a department physician, language barriers, and emergency procedures. Primary outcome of this study was the reduction of parental preprocedural anxiety, secondary outcomes included procedural understanding and satisfaction with the performed PE. The study design has been adapted from our previously described study.^8^ Briefly, parents took questionnaires prior to PE for baseline testing regarding patient characteristics, anxiety and procedural understanding. Custom-developed questionnaires (Supplement) were used for the evaluation of procedural understanding and satisfaction with the PE. Parental anxiety was evaluated using the Visual Analog Scale (VAS, 1-10) and the State-Trait Anxiety Inventory, made up of the State-Anxiety Inventory (STAI) and the Trait-Anxiety Inventory (TAI). The German short version of the STAI was used with 10 statements regarding the state anxiety and trait anxiety for the patient, respectively. Scores for each short inventory ranged from 10 to 40, while higher scores were associated with higher anxiety levels. The trait anxiety inventory of parents was collected before discharge.

To prevent investigator bias, the same physician conducted all PE sessions. After PE, parents immediately completed a second questionnaire, assessing procedural understanding, anxiety (using VAS and STAI), education quality, and satisfaction with the method. At discharge, parents filled out a final questionnaire to assess trait anxiety. Additionally, a brief questionnaire was distributed to physicians in pediatric cardiology and congenital cardiac surgery to evaluate their experiences with the education methods (Supplement).

### Model creation

**Fig. 2:**
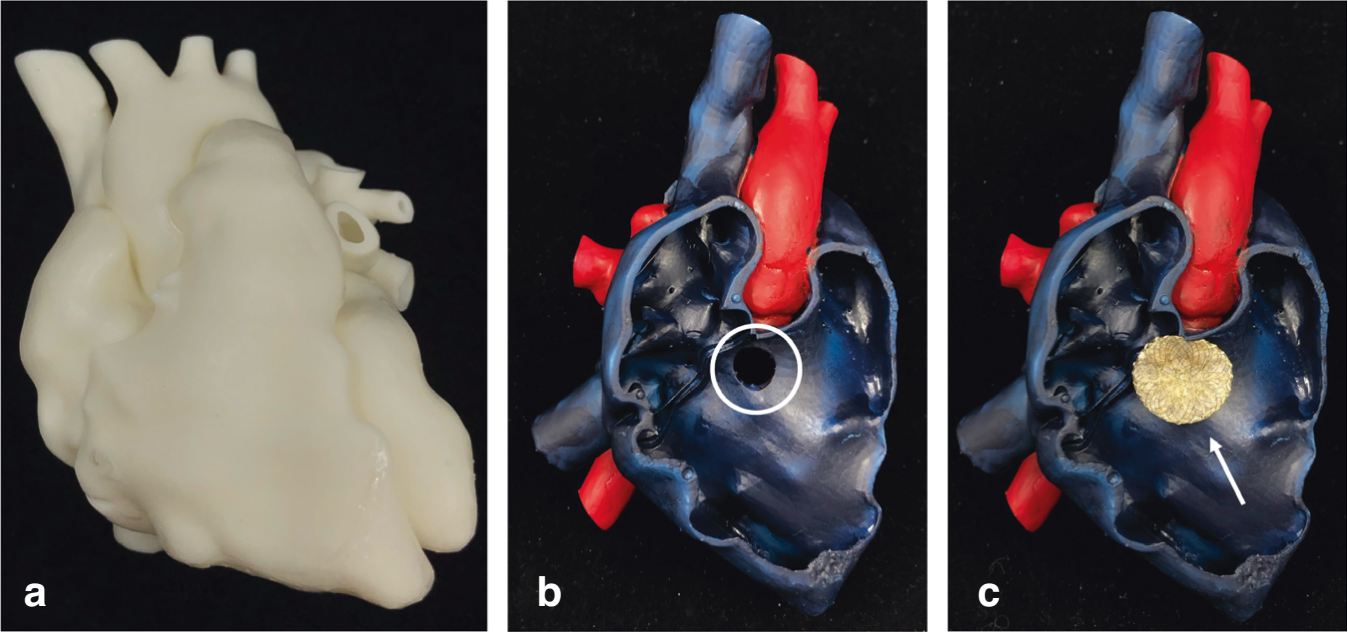
3D-printed models for CHD parental education. **a** 3D-printed hollow heart model using a white rigid material. **b** Color-painted and opened 3D-printed model visualizing a VSD (white circle) and **c** showing the exemplary therapeutic intervention with an occluder (white arrow).

Anonymized contrast-enhanced CT datasets with the corresponding pathologies were imported in Mimics Innovation Suite (Mimics 24.0, Materialise NV, Leuven, Belgium). After segmentation of the regions of interest, the models were 3D-printed using a rigid white material (White_v4, Formlabs Inc., Sommerville, MA) on a Formlabs Form 3 (Formlabs Inc.). Subsequently, the models were post processed according to manufacturer’s instructions and color-painted for better visualization (Fig. 2). One model of each of the following pathologies was segmented, 3D-printed and postprocessed: atrial and ventricular septal defect, aortic isthmus stenosis and patent ductus arterious. An atrial septal defect (ASD) occluder (Occlutech Holding AG, Schaffhausen, CHE) as well as a balloon catheter were used to demonstrate the procedural interventional steps.

### Statistical analysis

Analysis was performed using SPSS (v.29, IBM SPSS Statistics for Macintosh, IBM Corp., Armonk, NY) as well as GraphPad Prism Software (v.10.2.1, GraphPad Software, LLC, LA Jolla, CA). Categorical variables are presented as percentage of the total. Paired Student’s t-test was used for analysis following testing for normality with Shapiro-Wilk normality test. Multiple linear regression was performed to assess factors contributing to anxiety before and after the PE. *P* values < 0.05 were considered statistically significant.

## Results

Prior to parental enrollment, we conducted a short survey with pediatric cardiologists and congenital cardiac surgeons at LMU University Hospital and the German Heart Center in Munich. Physicians (*n* = 22) with a mean duration of professional experience of 14.86 years ( ± 8.99 years) reported to perform PE regarding CHD for interventions or surgeries about 3 times a week (2.89 ± 0.85 times). They need approximately 20 minutes for the PE of a cardiological intervention and 30 minutes for a congenital cardiac surgery. All participating physicians used standardized paper-based methods in addition to self-made drawings. Only 18.2% reported to use anatomical models regularly. Physicians unanimously agreed that new methods and additional models for PE, especially for complex procedures in the field of CHD, are necessary and long overdue, and found 3D-printed models to be contemporary.

Baseline characteristics of parents (*n* = 57, Table 1) showed a mean age of 38 years ( ± 9.00 years), 63% were female and every participant was previously informed and some (26.32%) had already undergone an intervention or surgery for CHD with their child before. The mean age of children was 1.7 years ( ± 3.94 years). The most common diagnosis was ASD, treated interventionally in 33.34% of cases and surgically in 22.81%. Surgical VSD repair was performed in 17.54% of cases. Other diagnoses included interventional therapy for PDA, interventional biopsy, and surgical repair for Fallot and CoA (Table 1). A total of 81% of parents had prior information from their pediatrician, and 54% had researched information online. There were no significant differences between the control and 3D model groups in baseline STAI and VAS scores, TAI scores, or the time spent on patient education.

**Table 1:** Baseline characteristics

Characteristic	Control (*n* = 28)	3D Model (*n* = 29)	Total (*n* = 57)
**Parents**			
Mean age years (SD)	38 (9.97)	39 (10)	38 (9)
Sex male	12 (42.85%)	12 (41.38%)	24 (42.11%)
Sex female	18 (64.28%)	18 (62.07%)	36 (63.16%)
**Child**			
Mean age years (SD)	3.28 (3.94)	4.59 (3.94)	1.7 (3.94)
Sex male	10 (35.71%)	13 (44.83%)	23 (40.36%)
Sex female	18 (64.28%)	17 (58.62%)	37 (64.91%)
**CHD**			
Interventional ASD	11 (39.29%)	8 (27.59%)	19 (33.34%)
Interventional PDA	6 (21.43%)	0	6 (10.53%)
Interventional Biopsy	0	3 (10.34%)	3 (5.26%)
Surgery ASD	7 (25%)	6 (20.70%)	13 (22.81%)
Surgery VSD	3 (10.71%)	7 (24.14%)	10 (17.54%)
Surgery Fallot	0	3 (10.34%)	3 (5.26%)
Surgery CoA	2 (7.14%)	2 (6.10%)	4 (7.02%)
Previously informed	28 (100%)	29 (100%)	57 (100%)
Previous interventions	9 (32.14%)	6 (20.69%)	15 (26.32%)
Cardiac catheterization	5 (17.86%)	0	5 (8.77%)
Catheterization + surgery	4 (14.29%)	6 (20.70%)	10 (17.54%)
Previous complications	0	1 (3.45%)	1 (1.75%)
Time for PE (SD)	25 (4)	25 (6)	25 (5)
STAI baseline (SD)	26 (6)	26 (6)	26 (6)
Trait-Anxiety-Score (SD)	20 (5)	19 (5)	19.5 (5)
Anxiety VAS baseline (SD)	6 (3)	6 (3)	6 (3)

Baseline characteristics of participants, data shown as percentages or mean ± standard deviation (SD) as shown below. *CHD* congenital heart disease, *ASD* atrial septal defect, *PDA* patent ductus arteriosus, *VSD* ventricular septal defect, *CoA* coarctation of the aorta, *PE* patient education, *STAI* State-Trait-Anxiety Inventory, *VAS* Visual Analog Scale.

**Fig. 3:**
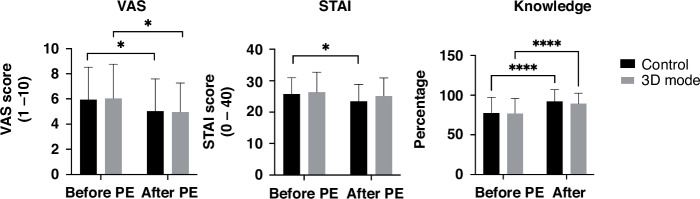
VAS and STAI score as well as knowledge gain. Data represented as mean ± standard deviation. *p **<** 0.05, **p **<** 0.005, ****p **<** 0.0001.

The control and 3D model group (Fig. 3) revealed a significant reduction in VAS anxiety after the PE (control: 5.9 to 5.0, ∆-0.9, *p* = 0.0342; 3D model: 6.0 to 4.9, ∆-1.1, *p* = 0.0116). The STAI score was significantly reduced after PE using paper-based methods and showed a noteworthy reduction, yet no statistically significant difference, after using the 3D models (control: 25.8 to 23.5, ∆-2.3, *p* = 0.009 and 3D model: 26.4 to 25.2, ∆-1.2, *p* = 0.0658). The analysis of the procedural knowledge gain after PE showed a highly significant increase in both groups (control: 77.5% to 92.3%; 3D model: 77% to 89,4%, *p* < 0.0001 each). Overall, participants of both groups were satisfied with the quality of the PE and rated a 3.6 ± 0.5 of a 4-point Likert scale.

After completion of data acquisition, multiple linear regression was used for analyzing contributing factors to anxiety levels prior to PE (VAS1) and after (VAS2) (Table 2). The regression analysis revealed statistically significant results for VAS1 [*R*^2^ = 0.78, *F* = (19, 36) = 6.689, *p* < 0.001]. Parents that presented with a medical background (4 nurses, 1 biologist) showed a lower VAS1 anxiety score (*B* = −0.232, *p* = 0.023) as well as parents that received additional and specific information from their pediatrician prior to the PE (*B* = −0.280, *p* = 0.008). Interestingly, parents that researched information online presented with an increased VAS1 anxiety score (*B* = 0.247, *p* = 0.013). A high STAI score before the PE (STAI1) led to the highest elevation of VAS1 anxiety (*B* = 0.753, *p* < 0.001).

**Table 2:** Analysis of anxiety scores before and after the PE.

Model		*B*	*SE*	*β*	*p*
1^a^	(Intercept)	−0.781	3.931		0.844
	Age parents	−0.016	0.033	−0.056	0.625
	Sex parents	0.906	0.566	0.168	0.118
	**Medical expertise**	−**1.830**	**0.772**	−**0.232**	**0.023**
	**Previously informed pediatrician**	−**1.777**	**0.638**	−**0.280**	**0.008**
	**Previously informed online**	**1.331**	**0.508**	**0.247**	**0.013**
	Previous cardiac procedure	−0.127	0.221	−0.058	0.569
	Previous complications	1.282	1.756	0.065	0.470
	VAS knowledge before PE	−0.246	0.148	−0.167	0.106
	**STAI1**	**0.346**	**0.045**	**0.753**	**<0.001**
	TAI	0.003	0.050	0.006	0.951
2^b^	(Intercept)	0.344	2.510		0.892
	Age parents	−0.008	0.022	−0.030	0.705
	Sex parents	0.280	0.352	0.054	0.432
	Medical expertise	−1.005	0.541	−0.132	0.071
	Previously informed pediatrician	0.096	0.452	0.016	0.832
	Previously informed online	0.667	0.414	0.128	0.115
	**Previous cardiac procedure**	−**0.381**	**0.149**	−**0.180**	**0.015**
	Previous complication	0.446	1.431	0.023	0.757
	STAI1	−0.025	0.056	−0.057	0.650
	**STAI2**	**0.330**	**0.051**	**0.725**	**<0.001**
	**VAS1**	**0.281**	**0.108**	**0.225**	**0.050**
	TAI	−0.63	0.036	−0.116	0.088

Analysis of contributing factors. Statistically significant values are in bold letters. Sex defined as *0* female, *1* male. *B* unstandardized coefficients, *SE* standard error, *β* standardized coefficients. VAS1 and STAI1 scores were collected prior to PE, VAS2 and STAI2 scores were collected after the PE. *TAI* Trait-Anxiety-Score.

^a^dependent Variable: VAS1.

^b^dependent Variable: VAS2.

Analysis of VAS anxiety score after PE (VAS2) revealed statistically significant results [*R*^*2*^ = 0.85, *F* = (15, 40), *p* < 0.001]. Parents that already underwent cardiac interventions or surgeries with their child showed a significant lower anxiety VAS2 score (*B* = −0.180, *p* = 0.015). Similar to the relation of VAS1 and STAI1, a higher STAI2 score led to a higher anxiety VAS2 score after PE (*B* = 0.725, *p* < 0.001).

## Discussion

The patient education is a crucial interaction between patients and their physicians and a legally required act before any medical intervention or surgery. Informed consent, particularly for parents of children with CHD, plays a significant role in the management of anxiety as well as the improvement of understanding complex medical procedures.^9^ Pediatric patients affected by CHD often require multiple interventions or surgeries with subsequent prolonged hospital stays. Previous studies have already demonstrated the substantial stress for parents of children with CHD. An effective PE can lead to a reduction of anxiety, mostly due to a better procedural understanding by the parents.^3,10^

In light of the current state-of-the-art, the results of the physicians’ questionnaire highlighted the need for improved visualization of complex anatomies to enhance physician-parent communication. All participating physicians reported supplementing their verbal explanations with hand-drawn illustrations to help explain the scheduled procedure when using standardized paper-based methods for parental education. Structured patient education has been shown to improve patients’ understanding of their condition, leading to better health behaviors and increased knowledge about the disease.^11^

Therefore, we evaluated the impact of the use of 3D-printed models on parental education efficacy as well as a possible reduction of anxiety. The currently standardized method using paper-based education constituted as a control group. Results revealed that 3D-printed cardiac models significantly reduced parental anxiety levels after patient education and increased knowledge about the disease and the imminent procedure.

The procedural knowledge gain was significantly increased after both methods of PE and the quality of the PE achieved very good and content ratings by the parents. Parents responded especially positive when educated using the 3D-printed cardiac models, as they stated in the evaluation form.

These findings are in accordance to previous studies that demonstrated a significant decrease in parental anxiety levels when educated additionally with cardiac models regarding cardiac surgery as well as other medical procedures.^6,12–14^ Anxiety levels, as measured by VAS, decreased after patient education in both the control and 3D model groups. Lower pre-education VAS anxiety scores were linked to a medical background and previous education provided by the pediatrician. Interestingly, parents who sought information online had higher anxiety levels before the educational session. While studies on parental health information-seeking behavior are limited, this factor is critical and should be considered by physicians during patient education [14]. Studies reveal that most parents rely on family and friends for additional information,^15^ yet they also use the internet and sometimes end up more anxious than before.^16^

Contradicting the results of decreased anxiety levels after the use of additional 3D-printed models, parents of children with CHD were found to be even more anxious after seeing 3D-printed models of patient-specific pathologies.^17^ This might be due to an enhanced and sudden understanding of the situation and underlying pathology with corresponding consequences of hospitalizations, helplessness and even financial burden. Parents’ psychological coping styles can also play an important role in the perception of medical information. As previously described by Miller, patients can either rather seek or avoid information about health threatening events.^18^ Naturally, parents of children with CHD can also display an increased interest in medical information or fall on the other side of the spectrum and rather avoid additional distress that may be caused by detailed medical information. This should be considered and be a focus of further analysis, as an adverse effect to 3D-printed models in such a patient group is of utmost clinical interest.

Parents in this study, however, who were already experienced with hospital stays of their children, showed a lower VAS anxiety score after the PE. It remains to be examined, if this is due to some kind of routine, higher mental resilience of these parents or better overall understanding of the situation and proceedings.

It is noteworthy, that the cohort of this study presented with elevated baseline anxiety levels at the beginning of the study compared to adult patients before cardiac surgery.^8^ Mean VAS at baseline was reported with 6 ± 3 (out of 10) and the State-Trait-Anxiety Score was elevated with 26 ± 5. These findings are consistent with previously reported elevated anxiety levels in parents of children with CHD.^19,20^ Despite contrary results and the absence of a highly significant reduction in anxiety, 3D-printed models will now constitute a standard for the support of physicians performing parental education on CHD procedures at our centers. We believe that the models enhance the procedural understanding more efficiently than a paper-based education alone and received very enthusiastic responses from affected parents.

We postulate that the high baseline anxiety of this cohort coupled with the level of experience regarding hospital proceedings led to only a slight reduction of anxiety and hence impact of 3D-printed models.

When interpreting the herein presented results, the limited sample size should be taken into consideration. Within the scope of the study, only parental anxiety was assessed. The pediatric patients’ anxiety was not evaluated, as the children were often too young to be interviewed.

Questions and evaluation of parental anxiety were conducted using standardized questionnaires which could be subject to bias. After consultation with the Department of Psychiatry of LMU Munich we used the State-Trait Anxiety Inventory as it is a highly standardized and validated assessment to prevent this bias. Tailored educational strategies considering parental background and taking previous parental experiences into account are recommended to optimize educational outcomes.

## Conclusion

This study evaluated the impact of 3D-printed models on reducing parental anxiety and enhancing procedural understanding in patients of children with CHD. Both, traditional paper-based methods and 3D-printed models, significantly improved procedural knowledge and reduced anxiety in parents. 3D-printed models were well-received and will be integrated into educational practices to enhance comprehension as well as physician-parent communication and optimal outcome.

## Supplementary information

checklist

Questionnaire No. 1

Questionnaire No. 2

Questionnaire No. 3

Questionnaire on previous educational experience of staff

## Data Availability

The datasets generated during and/or analyzed during the current study are available from the corresponding author on reasonable request.

## References

[1] Lindinger, A., Schwedler, G. & Hense, H. W. Prevalence of congenital heart defects in newborns in Germany: results of the first registration year of the pan study (july 2006 to june 2007). *Klin. Padiatr.* **222**, 321–326 (2010). 20665366 10.1055/s-0030-1254155

[2] Biber, S. et al. Current research status on the psychological situation of parents of children with congenital heart disease. *Cardiovasc Diagn. Ther.* **9**, S369–s376 (2019). 31737543 10.21037/cdt.2019.07.07 PMC6837930

[3] Boyer, P. J., Yell, J. A., Andrews, J. G. & Seckeler, M. D. Anxiety reduction after pre-procedure meetings in patients with Chd. *Cardiol. Young.* **30**, 991–994 (2020). 32500844 10.1017/S1047951120001407

[4] Kobayashi, D., Turner, D. R., Forbes, T. J. & Aggarwal, S. Parental anxiety among children undergoing cardiac catheterisation. *Cardiol. Young.* **28**, 315–321 (2018). 29081306 10.1017/S1047951117002074

[5] Lisanti, A. J. Parental stress and resilience in chd: a new frontier for health disparities research. *Cardiol. Young.* **28**, 1142–1150 (2018). 29991369 10.1017/S1047951118000963 PMC6103210

[6] Bernhard, J.-C. et al. Personalized 3d printed model of kidney and tumor anatomy: a useful tool for patient education. *World J. Urol.* **34**, 337–345 (2016). 26162845 10.1007/s00345-015-1632-2 PMC9084471

[7] Biglino, G. et al. Piloting the use of patient-specific cardiac models as a novel tool to facilitate communication during cinical consultations. *Pediatr. Cardiol.* **38**, 813–818 (2017). 28214968 10.1007/s00246-017-1586-9 PMC5388703

[8] Grab, M. et al. New perspectives in patient education for cardiac surgery using 3d-printing and virtual reality. *Front Cardiovasc Med*. **10**, 1092007 (2023). 36937915 10.3389/fcvm.2023.1092007 PMC10020687

[9] Werner, O., El Louali, F., Fouilloux, V., Amedro, P. & Ovaert, C. Parental anxiety before invasive cardiac procedure in children with congenital heart disease: contributing factors and consequences. *Congenit. Heart Dis.* **14**, 778–784 (2019). 31066183 10.1111/chd.12777

[10] Uzark, K. & Jones, K. Parenting stress and children with heart disease. *J. Pediatr. Health Care* **17**, 163–168 (2003). 12847425 10.1067/mph.2003.22

[11] Moons, P. et al. What do adult patients with congenital heart disease know about their disease, treatment, and prevention of complications? A call for structured patient education. *Heart* **86**, 74–80 (2001). 11410567 10.1136/heart.86.1.74 PMC1729797

[12] Biro, M. et al. The use of 3-dimensionally printed models to optimize patient education and alleviate perioperative anxiety in mohs micrographic surgery: a randomized controlled trial. *J. Am. Acad. Dermatol*. **81**, 1339–1345 (2019). 31163232 10.1016/j.jaad.2019.05.085 PMC7031844

[13] Marquess, M. et al. A pilot study to determine if the use of a virtual reality education module reduces anxiety and increases comprehension in patients receiving radiation therapy. *J. Radiat. Oncol.* **6**, 317–322 (2017).

[14] Karsenty, C. et al. Impact of 3d-printed models in meetings with parents of children undergoing interventional cardiac catheterisation. *Front Pediatr.* **10**, 947340 (2022). 36699296 10.3389/fped.2022.947340 PMC9869040

[15] Wong, M. K. Y., Sivasegaran, D., Choo, C. S. C. & Nah, S. A. Parental internet use and health information seeking behavior comparing elective and emergency pediatric surgical situations. *Eur. J. Pediatr. Surg.* **28**, 89–95 (2018). 28662533 10.1055/s-0037-1604021

[16] Nicholl, H., Tracey, C., Begley, T., King, C. & Lynch, A. M. Internet use by parents of children with rare conditions: findings from a study on parents’ web information needs. *J. Med. Internet Res*. **19**, e51 (2017). 28246072 10.2196/jmir.5834 PMC5350458

[17] Biglino, G. et al. 3d-manufactured patient-specific models of congenital heart defects for communication in clinical practice: feasibility and acceptability. *BMJ Open* **5**, e007165 (2015). 25933810 10.1136/bmjopen-2014-007165 PMC4420970

[18] Miller, S. M. Monitoring and blunting: validation of a questionnaire to assess styles of information seeking under threat. *J. Pers. Soc. Psychol.* **52**, 345–353 (1987). 3559895 10.1037//0022-3514.52.2.345

[19] Lee, B. K. & Loomba, R. S. Rates of Depression, Anxiety, and Stress in Parents of Children with Congenital Heart Disease Using the Depression Anxiety Stress Scale. *Ann. Pediatr. Cardiol.* **15**, 374–379 (2022). 36935826 10.4103/apc.apc_27_22 PMC10015400

[20] Woolf-King, S. E., Anger, A., Arnold, E. A., Weiss, S. J. & Teitel, D. Mental Health among Parents of Children with Critical Congenital Heart Defects: A Systematic Review. *J Am Heart Assoc* **6** (2017). 10.1161/JAHA.116.004862 PMC5523775 28151402

