# Supplementary material for: Evaluating 3D-printed models for congenital heart disease: impact on parental anxiety and procedural understanding
Source: Pediatr Res. 2025 Mar 17;98(5):1835–40. doi: 10.1038/s41390-025-03999-x (PMC12602363; doi:10.1038/s41390-025-03999-x)
Supplement: Supplementary file 2 — Questionnaire No. 1 [file 41390_2025_3999_MOESM2_ESM.pdf]

Questionnaire No. 1

Gender (parent)			Age (parent)
○ male	○ female	○ diverse	years

Gender (child)			Age (child)	
○ male	○ female	○ diverse	months	years

Do you have previous professional knowledge of the anatomy and function of the human heart?	
Yes:	Profession:
○ No	

Have you already informed yourself about the illness and the upcoming procedure before the training? (multiple answers possible)				
Yes:	○ Pediatrician/cardiologist	○ Literature	○ Internet	○ Other:
○ No				

Has your child already had heart surgery or a cardiac catheterization procedure?		
Yes:	○ Operation	○ Cardiac catheter
Complications	○ No	○ Yes
○ No		

Do you feel like you have control of the situation surrounding your child's illness?			
○ Not at all	○ Somewhat	○ Moderately so	○ Very much so

Questions about the training

How would you rate your knowledge of the procedure on a scale of 1 to 10? Please mark a number.									
1	2	3	4	5	6	7	8	9	10
no knowledge									a lot of knowledge

Please rate the following statements.	Not at all	Somewhat	Moderately so	Very much so	Prefer not to say
I understand the structure and function of the human heart.	○	○	○	○	○
I understand the cardiovascular system.	○	○	○	○	○
I understand my child's congenital heart defect.	○	○	○	○	○
I know in which part of the heart the congenital heart defect is located.	○	○	○	○	○
I understand the execution of the upcoming procedure.	○	○	○	○	○
I would like to be given detailed training about the upcoming procedure.	○	○	○	○	○
I can understand the positive effect of the procedure.	○	○	○	○	○
I feel confident enough to explain the procedure to a third person.	○	○	○	○	○

Questions about your mental state

How anxious do you feel at this moment on a scale of 1 to 10? Please mark a number?									
1	2	3	4	5	6	7	8	9	10
No fear									very strong fear

Please indicate how you feel about the following statements at this moment.	Not at all	Somewhat	Moderately so	Very much so
I am calm	○	○	○	○
I feel tense	○	○	○	○
I am excited	○	○	○	○
I feel rested	○	○	○	○
I am worried	○	○	○	○
I feel confident	○	○	○	○
I am nervous	○	○	○	○
I feel uptight	○	○	○	○
I am anxious	○	○	○	○
I am happy	○	○	○	○

Thank you for your participation.
