# Supplementary material for: Evaluating 3D-printed models for congenital heart disease: impact on parental anxiety and procedural understanding
Source: Pediatr Res. 2025 Mar 17;98(5):1835–40. doi: 10.1038/s41390-025-03999-x (PMC12602363; doi:10.1038/s41390-025-03999-x)
Supplement: Supplementary file 3 — Questionnaire No. 2 [file 41390_2025_3999_MOESM3_ESM.pdf]

Questionnaire No. 2

Questions about the training

Please rate the following statements.	Not at all	Somewhat	Moderately so	Very much so	Prefer not to say
I understand the structure and function of the human heart.	○	○	○	○	○
I understand the cardiovascular system.	○	○	○	○	○
I understand my child's congenital heart defect.	○	○	○	○	○
I know in which part of the heart the congenital heart defect is located.	○	○	○	○	○
I understand the execution of the upcoming procedure.	○	○	○	○	○
I found it helpful to be given detailed training about the upcoming procedure.	○	○	○	○	○
I can understand the positive effect of the procedure.	○	○	○	○	○
I feel confident enough to explain the procedure to a third person.	○	○	○	○	○

Please rate the following statements.	Not true at all	Rather not true	More likely	Totally true
The **training** helped improve my **understanding** of the **cardiovascular system**.	○	○	○	○
The **training medium** improved my **understanding** of the **cardiovascular system**.	○	○	○	○
The **training medium** helped me to **visualize** the **cardiovascular system** in **three dimensions**.	○	○	○	○
The **training** helped improve my **understanding** of the **heart disease**.	○	○	○	○
The **training medium** improved my **understanding** of the **heart disease**.	○	○	○	○
The **training medium** helped me to **visualize** the **heart disease** in **three dimensions**.	○	○	○	○
I can imagine the individual steps of the procedure better after the training than before.	○	○	○	○
I feel well trained about the upcoming procedure.	○	○	○	○
The person giving the training took enough time for the training.	○	○	○	○
I would recommend the training.	○	○	○	○

How would you rate your knowledge of the procedure on a scale of 1 to 10? Please mark a number.									
1	2	3	4	5	6	7	8	9	10
no knowledge									a lot of knowledge

How did the training influence your subsequent conditions?	Negative	Unchanged	Postitive
Anxiety	○	○	○
Restlessness	○	○	○
Hope	○	○	○
Trust in doctors	○	○	○
Trust in medicine	○	○	○

Please rate the training with a school grade!					
○ 1	○ 2	○ 3	○ 4	○ 5	○ 6

Do you have any suggestions for improving the training?

Do you have any suggestions for improving the training medium used?

Questions about your mental state

How anxious do you feel at this moment on a scale of 1 to 10? Please mark a number?									
1	2	3	4	5	6	7	8	9	10
No fear									very strong fear

Please indicate how you feel about the following statements at this moment.	Not at all	Somewhat	Moderately so	Very much so
I am calm	○	○	○	○
I feel tense	○	○	○	○
I am excited	○	○	○	○
I feel rested	○	○	○	○
I am worried	○	○	○	○
I feel confident	○	○	○	○
I am nervous	○	○	○	○
I feel uptight	○	○	○	○
I am anxious	○	○	○	○
I am happy	○	○	○	○

Notes by trainer. Please do not fill in.

Duration:
Training medium:
Comment:
