# Supplementary material for: Evaluating 3D-printed models for congenital heart disease: impact on parental anxiety and procedural understanding
Source: Pediatr Res. 2025 Mar 17;98(5):1835–40. doi: 10.1038/s41390-025-03999-x (PMC12602363; doi:10.1038/s41390-025-03999-x)
Supplement: Supplementary file 4 — Questionnaire No. 3 [file 41390_2025_3999_MOESM4_ESM.pdf]

Questionnaire No. 3

Questions about your mental state

Please indicate how you generally feel about the following statements.	Almost never	Sometimes	Often	Almost always
I get tired quickly.	○	○	○	○
I miss good opportunities because I can't decide quickly enough.	○	○	○	○
I am calm and relaxed.	○	○	○	○
I am happy.	○	○	○	○
I tend to take everything seriously.	○	○	○	○
I lack self-confidence.	○	○	○	○
I feel safe.	○	○	○	○
I feel depressed.	○	○	○	○
Unimportant thoughts go through my head and depress me.	○	○	○	○
I get nervous and restless when I think about my current affairs.	○	○	○	○

Since the procedure, have you suffered from:	Yes	no
Depressed mood	○	○
Loss of interest	○	○
Loss of drive	○	○

Are you currently suffering from diagnosed depression?	
○ Yes	○ No

Please note the back.

Did you feel like you had lost control of the situation surrounding your child’s illness?	
○	No
○	Yes, after making the diagnosis
○	Yes, after the patient education
○	Yes, during the procedure

When were you most worried about your child?	
○	I was not worried
○	After making the diagnosis
○	After the patient education
○	During the procedure

How anxious do you feel at this moment on a scale of 1 to 10? Please mark a number?									
1	2	3	4	5	6	7	8	9	10
No fear									very strong fear

Thank you for your participation.
