# Supplementary material for: Evaluating 3D-printed models for congenital heart disease: impact on parental anxiety and procedural understanding
Source: Pediatr Res. 2025 Mar 17;98(5):1835–40. doi: 10.1038/s41390-025-03999-x (PMC12602363; doi:10.1038/s41390-025-03999-x)
Supplement: Supplementary file 5 — Questionnaire on previous educational experience of staff [file 41390_2025_3999_MOESM5_ESM.pdf]

Questionnaire on previous educational experience of staff

Gender			Age
o male	o female	o diverse	years

How many years have you been working as a doctor?
years

What position do you have in the department?
o Resident
o Consultant
o Consultant in management position
Specialization: (multiple answers possible)
o Pediatrics
o Pediatric cardiology
o Cardiac surgery
o Pediatric cardiac surgery

How many patient educations do you conduct on average per week?

How would you rate your experience and routine with patient education on a scale of 1 to 10? Please mark a number.

1	2	3	4	5	6	7	8	9	10
Little experience/routine									lots of experience/routine

How much time does an average patient education take?
For cardiac surgery:
For cardiac catheterization:

Which educational media do you regularly use? (multiple answers possible)
o Standardized patient education form
o Hand-drawn sketch
o Models
o Videos
o Other:

Please note the back!

What advantages do you see in providing patient education through standardized patient education forms?

What disadvantages do you see in providing patient education through standardized patient education forms?

Would you like to have new media for patient education? (multiple answers possible)	
○	No
○	Yes, 3D models
○	Yes, virtual models (VR)
○	Yes, other:

Would you use new educational media even if it meant spending more time on your part?	
○ Yes	○ No

Thank you for your participation.
